# Co‐methylation network analysis of Psychosis in Alzheimer’s disease

**DOI:** 10.1002/alz.088982

**Published:** 2025-01-03

**Authors:** Morteza P Kouhsar, Luke Weymouth, Adam Smith, Jennifer Imm, Claudia Bredemeyer, Yehani Wedatilake, Ali Torkamani, Sverre Bergh, Geir Selbaek, Jonathan Mill, Clive G Ballard, Robert Sweet, Julia Kofler, Byron Creese, Ehsan Pishva, Katie Lunnon

**Affiliations:** ^1^ University of Exeter, Exeter United Kingdom; ^2^ University of Exeter, Exeter, Devon United Kingdom; ^3^ Norwegian National Advisory Unit on Aging and Health, Vestfold Hospital Trust, Tønsberg Norway; ^4^ Scripps Research, La Jolla, CA USA; ^5^ Centre for Old Age Psychiatric Research, Innlandet Hospital Trust, Ottestad Norway; ^6^ Oslo University Hospital, Oslo Norway; ^7^ Department of Psychiatry and Neurology, University of Pittsburgh, Pittsburgh, PA USA; ^8^ University of Pittsburgh, Pittsburgh, PA USA; ^9^ Brunel University London, London United Kingdom; ^10^ Maastricht University, Maastricht Netherlands

## Abstract

**Background:**

Psychosis (broadly delusions and hallucinations) has a cumulative disease prevalence of around 40% in Alzheimer’s disease (AD). The epigenomic, genomic, and neuropathological data provide powerful evidence that AD+P has a distinct neurobiological profile. Here, we used the weighted gene co‐expression network analysis (WGCNA) method to investigate DNA methylation associated with AD+P in the dorsolateral prefrontal cortex of 153 post‐mortem brain samples.

**Method:**

Our primary analysis focused on applying WGCNA to the PITT‐ADRC cohort, followed by subsequent replication of its findings in the BDR cohort. The genotype data from PITT‐ADRC and the WGCNA results were further utilized to identify the most significant methylation Quantitative Trait Loci (mQTLs) associated with psychosis. Subsequently, we explored RNA sequencing data from PITT‐ADRC to identify genes affected by the replicated findings uncovered in our primary analysis.

**Result:**

We identified five AD+P‐related modules in the PITT‐ADRC cohort, with one of them being replicated in the BDR cohort. This replicated AD+P‐related module exhibits a high enrichment in the T cell receptor signalling pathway. According to the colocalization analysis results, this module shares significant SNPs in some regions that are also significantly associated with Schizophrenia and Educational Attainment.

**Conclusion:**

Understanding molecular differences between AD and Psychosis at the genetic and epigenetic levels could guide us in discovering appropriate treatments for AD+P cases. To this end, we initiated a comprehensive, large sample‐sized network analysis study based on genetic and epigenetic data.

